# Spatial distribution characteristics of soil heavy metals in Sabao Chaqu watershed of Tuotuo river, Qinghai-Tibet Plateau based on geographic detector

**DOI:** 10.1038/s41598-023-48261-8

**Published:** 2024-04-22

**Authors:** Cang Gong, Changhai Tan, Hang Dong, Haichuan Lu, Shunxiang Wang, Zihong Liao, Duoji Wangzha, Wangdui Zhaxi, Jiancai Tudan, Lang Wen

**Affiliations:** 1 College of Earth and Environmental Sciences, Lanzhou University, Lanzhou, China; 2 Research Center of Applied Geology of China Geological Survey, Chengdu, China; 3 Special Fund of the National Key Laboratory of Water Disaster Prevention, NanJing, China; 4 Key Laboratory of Natural Resource Coupling Process and Effects, Beijing, China

**Keywords:** Biogeochemistry, Environmental sciences

## Abstract

The Qinghai-Tibet Plateau belongs to the area of extremely fragile environment and sensitive to human activities. In recent years, more and more human interference has been detected in this area. In this study, 128 surface soil samples were collected from the Sabao Chaqu watershed of the Tuotuo river at the source of the Yangtze River on the Qinghai-Tibet Plateau. The soil pollution status and spatial distribution characteristics of Cd, Hg, As, Cu, Pb, Cr, Zn and Ni were evaluated by soil accumulation index, enrichment factor, pollution index and geographical detector. The results showed that the average contents of As, Cd, Pb and Zn in the study area were 1.2–3.64 times higher than soil background values of Tibet, while the contents of Hg, Cr, Cu and Ni were lower than the background values, while the average content of As was higher than the soil pollution risk screening value (GB15618-2018), and the pollution index showed that As was in a low pollution state, while the other 7 heavy metals were in a safe state. There were significant differences in the spatial distribution of 8 heavy metals and there was a significant correlation with soil properties and distance factors. Factor detection showed that natural factors had the strongest explanatory power to the contents of As, Cd, Cr, Cu and Ni, distance from the lake and soil Sc content had the strongest explanatory power to Hg content, and anthropogenic factors had the strongest explanatory power to Pb content. Interaction detection revealed that the q values of the strongest interaction explanatory power for As, Cd, Cr, Cu, Hg, Ni, Pb, and Zn were 2.81, 4.30, 1.26, 2.47, 2.33, 1.59, 6.37, and 5.08 times higher than their strongest factor detection explanatory power, respectively. The interaction between anthropogenic factors and other factors has an important influence on the spatial differentiation of heavy metals in the study area. Risk detection showed that the average contents of As, Cd, Cr, Cu, Hg, Ni, Pb and Zn were the highest in the subregions of MgO, TS, Sc, X_6_, X_13_, MgO, TN and X_4_, respectively. Comprehensive study shows that the spatial differentiation of As, Cd, Cr, Cu, Ni and Zn is mainly affected by natural factors, but there are also some anthropogenic factors, the spatial differentiation of Hg is affected by both natural factors and atmospheric deposition, and the spatial distribution characteristics of Pb are mainly affected by anthropogenic factors.

## Introduction

Soil is the basis of all terrestrial ecosystems, an important place for the circulation of material and energy on the earth, and the basis of many ecosystem services related to the development of human society^1,2^. In addition, soil is an important recipient of various pollutants produced by human activities and is considered to be the largest sink of heavy metals on earth^3^. In recent years, the toxic pollution of heavy metals in soil has become a serious environmental problem in the world. The survey shows that the point exceeding rates of Cd, Hg, As, Cu, Pb, Cr, Zn, and Ni in the soil of China in 2014 were 7.0%, 1.6%, 2.7%, 2.1%, 1.5%, 1.1%, 0.9%, and 4.8%, respectively^4^. Heavy metals retained in soil not only pose a serious threat to living plants, soil animals and microorganisms, but also pose a potential threat to human health through the food chain^5,6^. Studies have shown that excessive intake of heavy metals can lead to a variety of chronic diseases, which pose risks to human health^7,8^. Effective treatment of soil heavy metal pollution tracing its source and exploring the factors affecting the distribution of heavy metals in soil are all key^4^. Therefore, in the past 10 years, a lot of research and analysis have been carried out on the driving factors of soil heavy metal pollution^9–14^.

Heavy metals in soil have two sources: natural and human activities^3,8^. The natural source of heavy metals is mainly due to rock weathering in the process of soil formation, and its concentration is usually harmless to the ecological environment^15,16^. Topography, altitude, geomorphology, climate and other natural factors affect the migration and transformation of heavy metals, which eventually lead to the spatial heterogeneity of heavy metals^17^. Various human activities such as industrial production, mineral mining and agricultural production will lead to the accumulation of heavy metals in the soil and aggravate the spatial variability^3,8,14,18^. In order to reduce soil pollution, reduce environmental risk, identify the main factors causing pollution, and remediate the soil at risk of heavy metal pollution, four problems must be identified first^19^: (1) Among the many influencing factors, which are the causes of pollution? (2) What is the degree of influence of these factors? (3) Do these influencing factors operate independently or are they related to each other? (4) What is the geographic scope of the pollution risk?

Multivariate statistical analysis, such as correlation analysis, principal component analysis, factor analysis, cluster analysis, regression analysis, and geostatistical analysis, such as spatial interpolation, spatial mapping and hot spot analysis, have been widely used to study the correlation between soil heavy metal pollution and pollution sources. However, based on the distribution characteristics of heavy metal elements, multivariate statistical analysis can be used to speculate the possible influencing factors, However, multivariate statistical analysis is based on the distribution characteristics of heavy metals and can be used to speculate on possible influencing factors, but it is not possible to determine the spatial distribution characteristics of these influencing factors^20^. Correlation analysis can be used to determine the quantitative relationship between the spatial distribution of soil heavy metals and influencing factors by cross-correlation map, but the interaction between influencing factors can’t be quantified^21^. Geostatistical analysis can be used to identify high-risk areas of pollution and analyze the contribution of different factors to spatial distribution characteristics^22^, but it cannot quantitatively measure the impact of each specific factor^23^. In addition, these methods require a relatively large number of samples for statistical inference^24^. In contrast, geographic detectors can reveal the influence of a single factor on dependent variables and the interaction of two factors without considering linearity and avoiding the influence of multivariable collinearity^25^. Geographic detectors include factor detection, interaction detection, risk area detection and ecological detection, which can quantitatively determine the effects of various factors on the spatial heterogeneity of soil heavy metals. The geographical detector can measure the contribution of various factors more intuitively, faster and more effectively, and there is no strong model hypothesis, which solves the limitations of traditional methods in analyzing category variables, and gradually gets better application results in the field of soil heavy metal pollution, which can effectively solve the above four problems^4,19,26–28^.

The Qinghai-Tibet Plateau, known as the “Roof of the World” or “the Third Pole”, is one of the areas least affected by human activities, under natural conditions, the content of heavy metals in the ecosystem of the Qinghai-Tibet Plateau is relatively low^29^. In recent years, with the climate change, the development and utilization of natural resources and the development of secondary and tertiary industries, the ecological environment of the Qinghai-Tibet Plateau has been gradually affected, and its soil system has been polluted by heavy metals to a certain extent^30^. In recent decades, the research results show that there are great differences in the sources of heavy metal elements in different research areas of the Qinghai-Tibet Plateau, including vehicles and other vehicles, the application of chemical fertilizers and pesticides, and a large number of exogenous pollutants, such as the deposition of atmospheric particles such as dust and aerosols outside the plateau^31–34^. Another important source of pollution is religious activities and large-scale sacrificial activities, which is the cause of slight pollution in non-industrial and remote areas^35,36^. In addition, the content of heavy metals transported by the Central Asian air mass and the Indian ocean sea air to the glacier snow on the Qinghai-Tibet Plateau is considerable. Under the influence of climate warming, these heavy metals are released into the river soil along with the melting water of ice and snow^36–38^. As the hinterland of the Qinghai-Tibet Plateau and the main basin at the source of the Yangtze River, the change characteristics of soil heavy metals in the Tuotuo river Basin have been concerned by domestic scholars. Liu^39^ found that compared with other rivers along the Yangtze River, the contents of Pb, Cd, Sb and Tl in the Tuotuo river were relatively higher, especially the Cd and Pb in its soil and surface sediments were enriched to varying degrees. The existing research results are of great significance for understanding the characteristics of heavy metals in the Qinghai-Tibet Plateau, but there are few reports on the research of heavy metals in the soil of the Tuotuo river basin at the source of the Yangtze River, especially the heavy metal content in the soil around the upper reaches of the Tuotuo river basin, Sabaochaqu basin.

Therefore, this study takes the Sabao Chaqu basin of the Tuotuo river at the source of the Yangtze River on the Qinghai-Tibet Plateau as the study area. Geographical detectors are used to carry out the following studies: (1) quantitatively calculate the influence degree of various factors on the spatial distribution of soil heavy metals; (2) determine the main factors affecting the spatial distribution of soil heavy metals; (3) analyze the interaction of various factors on the spatial distribution of soil heavy metals; (4) identify the risk areas with high risk of soil heavy metals. Clarify the distribution characteristics of soil heavy metal content in the study area, and then support and serve the construction of Tuotuo river Park in Sanjiangyuan National Park.

## Materials and methods

### Study area

**Figure 1:**
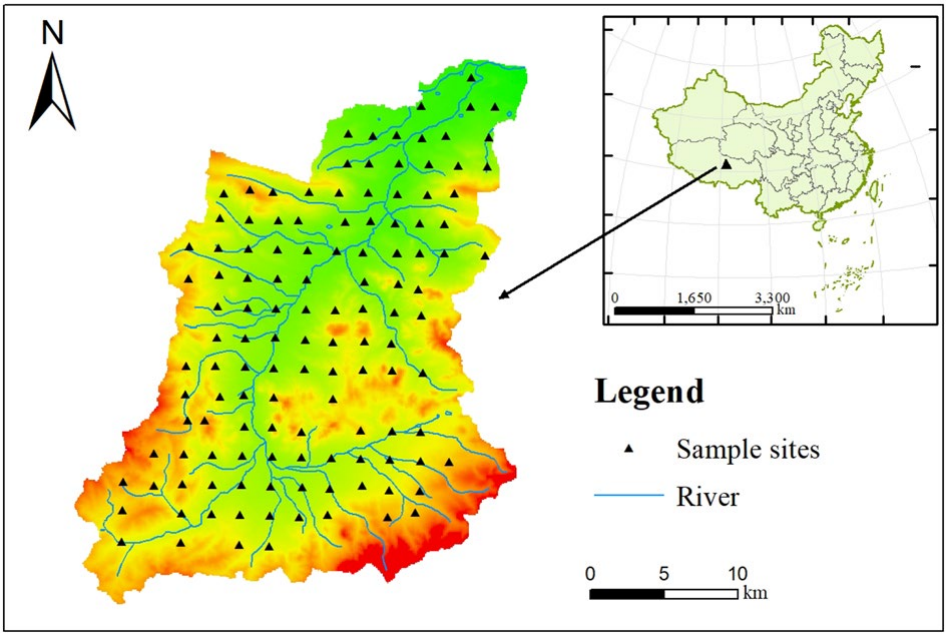
Locations of study area and sampling sites. (Map were generated with software ArcMap10.8 http://www.esri.com/).

The study area is located in the hinterland of the Qinghai-Tibet Plateau, the northernmost part of Anduo county, Tibet Autonomous region, the Hoh Xili reserve in Zhiduo county, Qinghai province in the north, and Yanshiping town in Anduo county in the east (90° 32° 47.62ʹʹ–91° 49° 13.06ʹʹ E, 33° 23° 16.46ʹʹ–34° 41° 31.47ʹʹ N) (Fig. 1). The climate belongs to the excessive zone of cold, semi-arid and semi-humid climate, which is an alpine steppe ecosystem with cold and dry, thin air, strong wind, open terrain, and high wind speed under the influence of cold air activity near the ground and strong westerly wind from the sky. The annual average number of gale days is more than 110 days. The temperature and pressure are low, the temperature difference between day and night is large, and the radiation is strong. The freezing period is from September to April of the following year. The annual average pressure is 584.3 Mb, and the annual average temperature is − 4.2 °C. The climate in the basin is dry and cold, the precipitation is less, and the natural environment is bad. More than 90% of the area belongs to no man’s land.

### Sampling and analysis

Field sampling will be completed in 2022. A total of 128 pieces of topsoil (0–20 cm) were collected according to the 1:250,000 land quality geochemical evaluation specification. The sampling locations are shown in Fig. 1. In order to improve the representativeness of soil samples, the sampling points were uniformly arranged in a 4 km^2^ sampling grid, and the distance between each sampling point was required to be greater than 2 km. 3–5 multi-point collections within 100 m around the sampling point are combined into one sample, and the original weight of the combined sample is greater than 1 kg. Locate sampling points with portable GPS. Visible impurities were removed from all collected samples and then air-dried at room temperature. The analysis and testing were completed in 2022 by Chengdu Comprehensive Rock and Mineral Testing Center of Sichuan Provincial Bureau of Geology and Mineral Exploration and Development. pH is measured by ion selective electrode method, TOC is measured by volumetric method, TN is measured by combustion infrared method, As and Hg are measured by atomic fluorescence method, Cu, Pb, Zn, Ni, Cr, Cd, TP and TK are measured by X-ray Fluorescence, inductively coupled plasma light/mass spectrometry. The quality of analysis and test was controlled by means of inserting national level soil standard material, repeatability inspection, anomaly inspection and blank test. The test quality parameters all meet the standard requirements, and the result data are real and reliable.

### Geoaccumulation index (I_geo_)

The geoaccumulation index (I_geo_) method can be used to compare the concentration of different heavy metals in soil and their pollution degree^19^.

1 $${I}_{geo}=\mathrm{ log}2\left(\frac{{C}_{i}}{K{\times B}_{i}}\right),$$

where I_geo_ is the soil accumulation index of heavy metal *i*; C*i* is the measured value of soil heavy metal *i*; B*i* is the reference value, and the soil background value of Qinghai-Tibet Plateau is selected; k is the correction coefficient, generally 1.5. The pollution degree of *I*_*geo*_ can be divided into seven grades: I_geo_ < 0, 0 ≤ I_geo_ < 1, 1 ≤ I_geo_ < 2, 2 ≤ I_geo_ < 3, 3 ≤ I_geo_ < 4, 4 ≤ I_geo_ < 5 and I_geo_ ≤ 5 correspond to unpolluted, mild polluted, moderate polluted, moderate-heavy polluted, heavy polluted, heavy-extreme polluted and extremely heavy polluted, respectively.

### Enrichment factor (EF)

The enrichment factor (EF) is a useful index to distinguish between natural and anthropogenic sources of heavy metals. EF can be calculated based on the following functions^40^:

2 $$ {\text{EF}} = \left[ {{\text{M}}_{i} /{\text{M}}_{Sc} } \right]_{{\text{S}}} /\left[ {{\text{M}}_{i} /{\text{M}}_{Sc} } \right]_{{\text{B}}} , $$

where [M_*i*_/M_*Sc*_]_S_ is the concentration ratio of the heavy metal *i* to Sc in samples, while [M_*i*_/M_*Sc*_]_B_ is the ratio of background values. Sc is a trace element, and has no significant anthropogenic sources, so Sc is chosen as the reference element^40^. Generally, according EF value the soils can be classified as deficiencyto minimal enrichment (< 1), mild enrichment (1–2), moderate enrichment (2–5), significant enrichment (5–20), very high enrichment (20–40), or extremely high enrichment (≥ 40)

### Pollution index (PI) and synthetic pollution index (SPI)

In order to assess the level of HMs pollution in the soil, a single factor PI and SPI were calculated:

3 $$\mathrm{PI }= \frac{{C}_{i}}{{S}_{i}},$$

4 $$\mathrm{SPI }= \sqrt{\frac{{\left(\frac{{C}_{i}}{{S}_{i}}\right)}_{max}+{\left(\frac{{C}_{i}}{{S}_{i}}\right)}_{ave}}{2}},$$

where PI is the pollution index of element *i* and SPI is the synthetical score of each heavy metals to the composite pollution. S_*i*_ is the evaluation standard of the *i* element, and the national control thresholds were chosen as the standard (Table 1). There are five pollution categories based on PI and SPI values: < 0.7, 0.7–1, 1–2, 2–3, ≥ 3, representing safety, alert, low pollution, moderate pollution, and severe pollution, respectively^41^.

**Table 1:** Statistical results of discretization of influence factors.

Factor	Unit	L1	L2	L3	L4	L5	L6	L7	L8	L9	L10
SiO_2_	%	< 49.14	49.14–53.8	53.8–56.24	56.24–58.29	58.29–60.38	60.38–61.95	61.95–64	64–66.95	66.95–70.88	> 70.88
Al_2_O_3_	%	< 8.05	8.05–8.86	8.86–9.35	9.35–9.73	9.73–10.3	10.3–10.9	10.9–11.9	11.9–13.1	13.1–14.6	> 14.6
CaO	%	< 2.32	2.32–3.23	3.23–4.32	4.32–5.18	5.18–5.92	5.92–6.48	6.48–7.87	7.87–10.28	10.28–13.92	> 13.92
TFe_2_O_3_	%	< 2.26	2.26–2.58	2.58–2.8	2.8–2.99	2.99–3.22	3.22–3.54	3.54–3.95	3.95–4.49	4.49–5.42	> 5.42
K_2_O	%	< 1.46	1.46–1.74	1.74–1.98	1.98–2.18	2.18–2.36	2.36–2.57	2.57–2.88	2.88–3.28	3.28–3.99	> 3.99
MgO	%	< 0.677	0.677–0.84	0.84–0.928	0.928–1.03	1.03–1.22	1.22–1.43	1.43–1.64	1.64–1.99	1.99–2.46	> 2.46
Na_2_O	%	< 0.73	0.73–0.948	0.948–1.13	1.13–1.31	1.31–1.49	1.49–1.73	1.73–1.98	1.98–2.36	2.36–2.9	> 2.9
TC	%	< 0.87	0.87–1.32	1.32–1.62	1.62–1.9	1.9–2.13	2.13–2.37	2.37–2.77	2.77–3.18	3.18–3.65	> 3.65
C_org_	%	< 0.41	0.41–0.59	0.59–0.68	0.68–0.78	0.78–0.91	0.91–1.09	1.09–1.26	1.26–1.5	1.5–1.86	> 1.86
TN	mg/kg	< 502	502–680	680–808	808–892	892–981	981–1132	1132–1243	1243–1394	1394–1739	> 1739
TP	mg/kg	< 459	459–521	521–577	577–623	623–668	668–719	719–794	794–926	926–1104	> 1104
TS	mg/kg	< 169	169–200	200–228	228–252	252–278	278–311	311–350	350–407	407–504	> 504
Sc	mg/kg	< 4.91	4.91–5.58	5.58–6.02	6.02–6.62	6.62–7.24	7.24–8.07	8.07–9.23	9.23–10.6	10.6–12.6	> 12.6
pH	–	< 8.11	8.11–8.36	8.36–8.47	8.47–8.56	8.56–8.64	8.64–8.73	8.73–8.82	8.82–8.98	8.98–9.14	> 9.14
NDVI	–	< 0.12	0.12–0.17	0.17–0.21	0.21–0.25	0.25–0.29	0.29–0.33	0.33–0.37	0.37–0.41	0.41–0.47	> 0.47
X_1_	m	< 4769	4769–4806	4806–4847	4847–4893	4893–4922	4922–4948	4948–4986	4986–5023	5023–5065	> 5065
X_2_	°	< 0.7	0.7–1.5	1.5–2.4	2.4–3.1	3.1–4	4–5.5	5.5–6.8	6.8–8.9	8.9–11.4	> 11.4
X_3_	°	< 23.4	23.4–46.8	46.8–76.8	76.8–106	106–154.4	154.4–201.7	201.7–249	249–288.9	288.9–323.6	> 323.6
X_4_^a^	–	Residual PM	Residual and wind-blown PM	Residual and slope PM	Alluvial PM	Alluvial and diluvial PM	Wind-blown PM	Diluvial PM	Diluvial and wind-blown PM	Slope PM	Slope and alluvial PM
X_5_	–	Meadow swamp soil	Grassland sandy soil	Alpine meadow grassland soil	Alpine frost desert soil	Alpine desert grassland soil	Alpine desert soil	Alpine wet meadow soil	Newly accumulated soil	–	–
X_6_	–	No erosion	Mild erosion	Moderate erosion	Severe erosion	–	–	–	–	–	–
X_7_	m	< 77,397	77,397–81,118	81,118–83,831	83,831–85,383	85,383–86,820	86,820–88,356	88,356–89,613	89,613–91,195	91,195–93,212	> 93,212
X_8_	m	< 76,893	76,893–80,849	80,849–83,097	83,097–85,329	85,329–86,902	86,902–88,426	88,426–89,844	89,844–91,566	91,566–93,544	> 93,544
X_9_	m	< 21,469	21,469–25,424	25,424–27,658	27,658–29,349	29,349–30,861	30,861–32,077	32,077–33,347	33,347–35,169	35,169–36,813	> 36,813
X_11_	m	< 443	443–1386	1386–1955	1955–2533	2533–3135	3135–3857	3857–4703	4703–5787	5787–7085	> 7085
X_12_	m	< 22,890	22,890–26,551	26,551–29,430	29,430–31,677	31,677–33,347	33,347–34,850	34,850–36,278	36,278–37,893	37,893–39,789	> 39,789
X_13_	m	< 619	619–1457	1457–2067	2067–2482	2482–2964	2964–3581	3581–4388	4388–5356	5356–7103	> 7103
X_14_	m	< 90	90–198	198–329	329–470	470–592	592–751	751–940	940–1196	1196–1868	> 1868

^a^*PM* parent materia; elevation (X_1_), slope (X_2_), aspect (X_3_), soil parent materials (X_4_), soil types (X_5_), soil erosion degree (X_6_), distance from railway (X_7_), distance from national highway G109 (X_8_), distance from county road (X_9_), distance from pastoral point (X_11_), distance from rural area (X_12_), distance from lake (X_13_), distance from river (X_14_).

### Geographical detector

Geographic detector measure the contribution of independent variables to dependent variables by calculating the ratio of the sum of the variances of the respective variables after classification to the sum of the variances of the dependent variable, including factor detectors, interaction detectors, risk detectors, and ecological detectors^25^.

Factor detector: used to detect the spatial differentiation of dependent variables and the ability of their respective variables to explain the influence of dependent variables, measured by the value of *q*:

5 $$q=1-\frac{\sum_{h=1}^{L}{N}_{h}{\sigma }_{h}^{2}}{N{\sigma }^{2}}=1-\frac{SSW}{SST},$$

where *h* = 1,…, L is the classification number of the independent variable X, N_h_ and N are the classification h and the number of units in the whole region, ${\sigma }_{h}^{2}$ and ${\sigma }^{2}$ the variance of the dependent variable Y in the classification *h* and the region, respectively. SSW and SST represent the sum of the variances of all categories of the independent variable X and the total variance in the region, respectively. The range of *q* is [0,1]. The larger the value of *q* is, the greater the influence of the independent variable X on the dependent variable Y is.

*Interaction detector* by identifying the *q* value of the interaction between two different independent variables, the influence of the interaction between independent variables on the dependent variable is judged on the basis of: when *q*(X_1_ ∩ X_2_) < min(*q*(X_1_), *q*(X_2_)), the interaction decreases nonlinearly; when min(*q*(X_1_),* q*(X_2_)) < *q*(X_1_ ∩ X_2_) < max(*q*(X_1_), *q*(X_2_)), it is a single factor nonlinear weakening; when *q*(X_1_ ∩ X_2_) > max(*q*(X_1_), *q*(X_2_)) is a double factor enhancement; when *q*(X_1_ ∩ X_2_) = *q*(X_1_) + *q*(X_2_), it is an independent interaction; when *q*(X_1_ ∩ X_2_) > *q*(X_1_) + q(X_2_) is nonlinear enhancement.

*Risk detector* it is mainly used to detect whether the influence factors are at risk to soil heavy metals, and *t* statistics are used to test it.

6 $${t}_{{\overline{y} }_{h=1}-{\overline{y} }_{h=2}}=\frac{{\overline{Y} }_{h=1}-{\overline{Y} }_{h=2}}{{\left[\frac{Var({\overline{Y} }_{h=1})}{{n}_{h=1}}+\frac{Var({\overline{Y} }_{h=2})}{{n}_{h=2}}\right]}^{1/2}},$$

where ${\overline{Y} }_{h}$ represents the mean value of attributes in sub-region *h*, in this study, the content of heavy metal elements; *Var* represents variance; *n*_*h*_ is the number of samples in sub-region *h*; the statistic *t* approximately obeys Student’s *t* distribution, and the higher the* t* value, the greater the influence of the influence factor on the spatial differentiation of soil heavy metals.

*Ecological detector* it is used to compare whether there is a significant difference between the two factors on the spatial distribution of soil heavy metals, which is measured by F statistics.

7 $$F=\frac{{SSW}_{X1}{N}_{X1}\left({N}_{X2}-1\right)}{{SSW}_{X2}{N}_{X2}\left({N}_{X1}-1\right)},$$

8 $${SSW}_{X1}=\sum_{h=1}^{L1}{N}_{h}{\sigma }_{h}^{2}, {SSW}_{X2}=\sum_{h=1}^{L2}{N}_{h}{\sigma }_{h}^{2},$$

where N_X1_ and N_X2_ represent the sample size of two independent variables X_1_ and X_2_ respectively; *SSW*_*X1*_ and *SSW*_*X2*_ represent the sum of intra-layer variances formed by X_1_ and X_2_, respectively; and L_1_ and L_2_ represent the number of variables X_1_ and X_2_, respectively. Where zero assumes H0: SSW_X1_ = SSW_X2_. If H0 is rejected at the significance level of *α*, it shows that there is a significant difference in the influence of two independent variables X_1_ and X_2_ on the spatial distribution of attribute dependent variable Y.

### Factor index selection and data processing

Referring to the selection methods of other scholars’ factor indicators, combined with the actual situation of the study area. Select soil properties (SiO_2_, Al_2_O_3_, CaO, TFe_2_O_3_, K_2_O, MgO, Na_2_O, total carbon (TC), organic carbon (C_org_), total nitrogen (TN), total phosphorus (TP), total sulfur (TS), Sc and pH), normalized vegetation cover index (NDVI), topographic factors (elevation (X_1_), slope (X_2_), aspect (X_3_)), soil parent materials (X_4_), soil types (X_5_), soil erosion degree (X_6_), distance factor (distance from railway (X_7_), distance from national highway G109 (X_8_), distance from county road (X_9_), distance from pastoral point (X_11_), distance from rural area (X_12_), distance from lake (X_13_), distance from river (X_14_)) 28 factors. Elevation data (GDEMDEM30m) comes from geospatial data cloud (http://www.gscloud.cn). Because when using geographic detector to analyze the influencing factors, the dependent variable must be a numerical variable, the independent variable must be a type variable, and if the independent variable is a numerical variable, it needs to be discretized into type variables. In this study, the natural breakpoint method is used to classify the influencing factors, and the classification results are shown in Table 1. Descriptive statistical analysis and correlation analysis of the data are carried out by SPSS26.0, sampling map and spatial distribution map are drawn by ArcGIS10.8, mapping is completed by Origin2019, and geographic detector is completed by GeoDetector software (http://www.geodetector.org/).

## Results and discussion

### Basic properties of topsoil in the study area

The contents and physicochemical properties of heavy metals in topsoil in the study area are shown in Table 2. The soils of all sampling sites are alkaline (pH > 7.5), the range of soil pH is 8.02–10.3, the average value is 8.67, higher than the background value of soil pH in Tibet and the geochemical baseline values of soil in Lhasa^42,43^. The mean concentrations of SiO_2_, Al_2_O_3_, CaO, TFe_2_O_3_, K_2_O, MgO, Na_2_O, C, C_org_, N, P, S, Sc were 60.5%, 11.0%, 6.36%, 3.39%, 2.48%, 1.28%, 1.52%, 2.26%, 0.96 mg/kg, 997 mg/kg, 727 mg/kg, 297 mg/kg and 7.70 mg/kg. The average values of soil As, Cd, Cr, Cu, Hg, Ni, Pb and Zn are 32.0, 0.29, 66.0, 17.3, 0.021, 27.8, 49.2 and 88.5 mg/kg, respectively. The contents of As, Cd, Pb and Zn in the study area were significantly higher than the background values of Tibetan soils^42^, and the contents of heavy metals except Cu were higher than the geochemical baseline values of soil in Lhasa^43^. Many studies have pointed out that the coefficient of variation is proportional to the degree of interference from external factors such as human activities^4^. The high coefficient of variation of As, Cd, Pb and Zn in the study area indicates that there are great differences in their contents in different sampling sites, indicating that they may be affected by some external interference factors. Considering that atmospheric circulation is one of the most common ways for heavy metals to enter the terrestrial ecosystem of Tibet, the increase in the concentration of As, Cd, Pb and Zn in the study area may be attributed to the long-distance transport of heavy metals in the surrounding area^44^. The average content of As in the soil was higher than the soil pollution risk screening value (GB15618-2018), while the average contents of Cd, Cr, Cu, Hg, Ni, Pb and Zn were significantly lower than the soil pollution risk screening value.

**Table 2:** Descriptive statistical results of soil composition.

Constituent	Unit	Min	Max	Mean	S.D.	Median	CV (%)	Geochemical baseline values of soil in Lhasa^43^	Background values of soil in Tibet^42^	Threshold values^a^ (pH > 7.5)
As	mg/kg	10.2	157	32.0	23.1	25.1	72.2	20	19.7	25
Cd	mg/kg	0.08	2.18	0.29	0.24	0.25	79.8	0.13	0.081	0.60
Cr	mg/kg	41	110	66.0	11.9	65.9	18.0	42	76.6	250
Cu	mg/kg	8.71	87.2	17.3	7.77	16.5	44.8	23	21.9	100
Hg	mg/kg	0.010	0.066	0.021	0.0072	0.020	33.5	0.079	0.024	3.4
Ni	mg/kg	12.1	48.4	27.8	7.51	26.9	27.0	21	32.1	190
Pb	mg/kg	17.9	584	49.2	52.0	40.7	106	31	29.1	170
Zn	mg/kg	38.3	582	88.5	57.7	75.7	65.2	70	74	300
SiO_2_	%	43.2	74.7	60.5	6.05	60.8	10.0	65.08	–	–
Al_2_O_3_	%	7.23	16.4	11.0	1.83	10.8	16.6	13.74	–	–
CaO	%	0.44	18.1	6.36	3.06	5.78	48.0	2.24	–	–
TFe_2_O_3_	%	2.02	6.49	3.39	0.84	3.30	24.9	3.91	–	–
K_2_O	%	1.24	4.84	2.48	0.75	2.33	30.1	3	–	–
MgO	%	0.53	3.17	1.28	0.43	1.27	33.7	1.18	–	–
Na_2_O	%	0.38	3.55	1.52	0.62	1.38	41.2	2.07	–	–
C	%	0.61	4.41	2.26	0.78	2.13	34.4	0.88	–	–
C_org_	%	0.28	2.84	0.96	0.42	0.89	43.4	0.72	2.68	–
N	mg/kg	320	2476	997	341	945	34.2	805	–	–
P	mg/kg	381	1521	727	228	671	31.3	862	–	–
S	mg/kg	149	680	297	94.2	283	31.7	225	–	–
Sc	mg/kg	3.92	16.1	7.70	2.29	7.42	29.8	9	10.2	–
pH	–	8.02	10.3	8.64	0.25	8.62	2.9	8.3	7.6	–

^a^The risk screening values for soil contamination (GB 15618-2018).

### Spatial distribution characteristics of soil heavy metals

**Figure 2:**
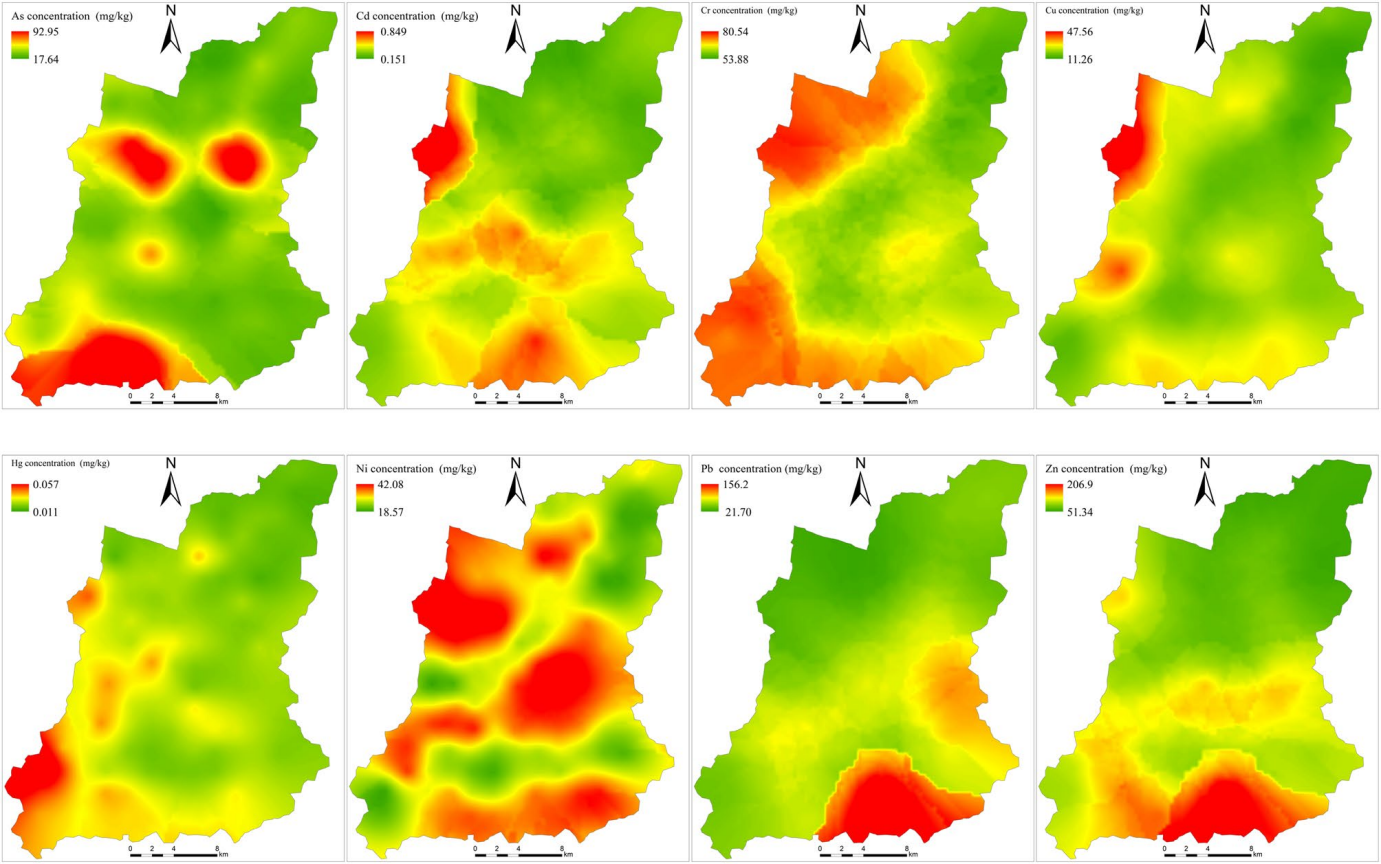
Spatial distribution of the heavy metals in the topsoil. (Map were generated with software ArcMap10.8 http://www.esri.com/).

Figure 2 shows the spatial distribution of 8 heavy metals in the soil of the study area. It can be seen that the high value areas of As are distributed in the southern and central regions, the high value areas of Cd are distributed in the west and south regions, the high value areas of Cr are distributed in the northwest and southwest regions, the high value areas of Cu are mainly distributed in a few regions in the west, the high value areas of Hg are mainly distributed in the southwest, the high value areas of Ni are concentrated in the northwest, central and southern regions, and the high value areas of Pb and Zn are mainly concentrated in the southern region.

### Evaluation of soil heavy metals pollution

**Figure 3:**
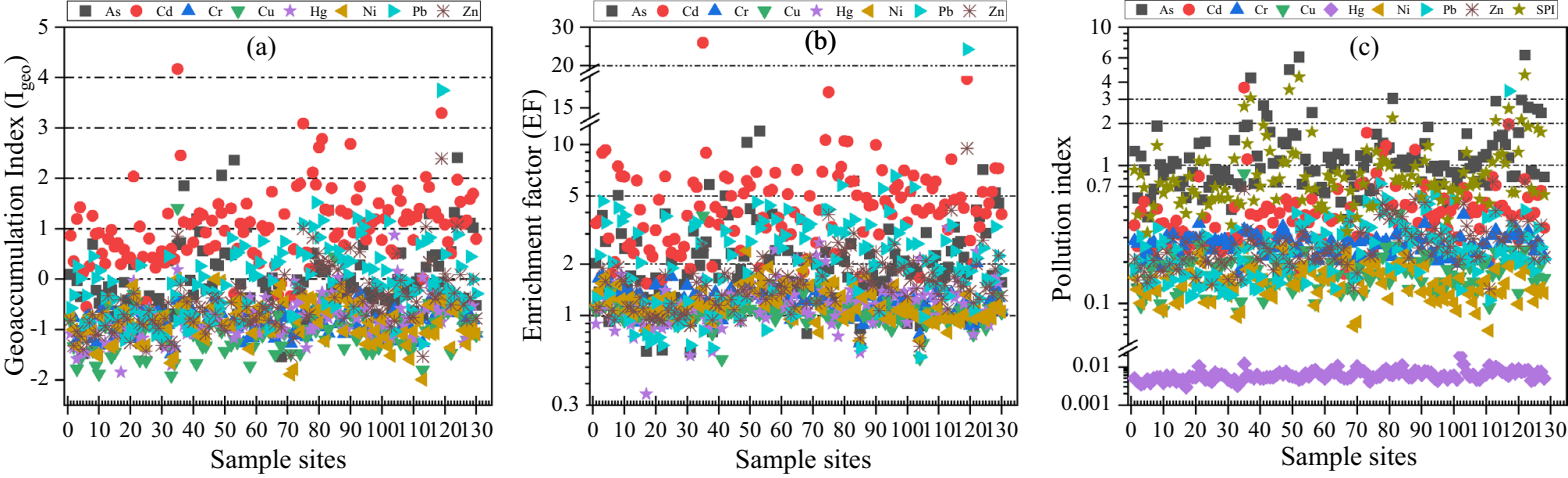
Scatter diagram for geoaccumulation index (I_geo_), enrichment factor (EF), and pollution index for As, Cd, Cr, Cu, Hg, Ni, Pb and Zn in this study.

Based on I_*geo*_ (Fig. 3a), the content of Cr in all samples was unpolluted; 98.44% of the samples were unpolluted with Cu and Ni, and 1.56% of the samples were mild to moderate polluted; for As, 67.97% of the samples was unpolluted, but 22.66%, 7.03% and 2.34% of the samples were mild, moderate and moderate-heavy polluted, respectively; for Cd, only 4.69% of the samples were unpolluted, but 43.75%, 43.75%, 5.47%, 1.56% and 0.78% were mild, moderate, moderate-heavy polluted, heavy polluted, respectively; for Hg, 96.09% of the samples were unpolluted, and 3.91% of the samples were mild polluted; for Pb, 58.59% of the samples were unpolluted, 34.38% and 6.25% of the samples were mild and moderate polluted; the Zn content in 84.38% of the samples was unpolluted, but 13.28%, 1.56% and 0.78% of the samples were mild, moderate and moderate-heavy polluted, respectively.

Based on EF (Fig. 3b), Cr, Cu, Hg, and Ni of the samples exhibit similar enrichment phenomena, with 18.75%, 38.28%, 24.22%, and 25.00% had minimal enrichment, 80.47%, 60.94%, 69.53%, and 73.44% had mild enrichment, and 0.78%, 0.78%, 6.25%, and 1.56% had moderate enrichment, respectively; for As and Zn in the sample, 9.38% and 10.94% had minimal enrichment, 48.44% and 74.22% had mild enrichment, 35.94% and 14.06% a had moderate enrichment, and 6.25% and 0.78% had significantly enrichment, respectively; for Pb, 17.19%, 32.03%, 46.88%, 3.13% and 0.78% of samples had minimal enrichment, mild enrichment, moderate enrichment, significant enrichment and very high enrichment, respectively; all the samples had different degrees of Cd enrichment, and 7.03%, 55.47%, 36.72% and 0.78% of the samples had mild enrichment, moderate enrichment, significant enrichment and very high enrichment, respectively.

Based on PI (Fig. 3c), 100% of the samples tested for Cr, Hg and Ni content are safety; for Cu, 99.22% samples are safety, 0.78% are on alert; for Cd and Pb, 86.72% and 98.44% are safety, 7.81% and 0.79% are alert, and 0.78% are severe pollution; for Zn, the samples in safety, alert and low pollution accounted for 96.88%, 2.34% and 0.78% respectively; for As, the samples in safety, alert, low pollution, moderate pollution and severe pollution accounted for 14.06%, 35.94%, 39.84%, 6.25% and 3.91%, respectively. From the SPI, the samples with safety, alert, low pollution, moderate pollution and severe pollution are 42.19%, 28.91%, 21.88%, 3.91% and 3.13%, respectively.

Generally, the I_*geo*_ and EF of Cd, As, Pb and Zn in the study area were significantly higher than Hg, Cr, Ni and Cu. The samples with moderate and above pollution (I_*geo*_ ≥ 2) of Cd, As, Pb and Zn accounted for 49.22%, 9.38%, 6.25% and 2.34%, respectively, and the samples with moderate and above enrichment (EF ≥ 2) of Cd, As, Pb and Zn accounted for 92.97%, 42.19%, 50.78% and 14.84%, respectively. On the one hand, it is related to the release of heavy metals in the diagenetic process of the study area, and it also means that there may be some external sources of heavy metals in the soil of the study area. SPI results show that 28.92% of the samples in the study area are in low pollution and above, and the environmental state varies from low pollution to serious pollution.

### Relativity analysis

**Figure 4:**
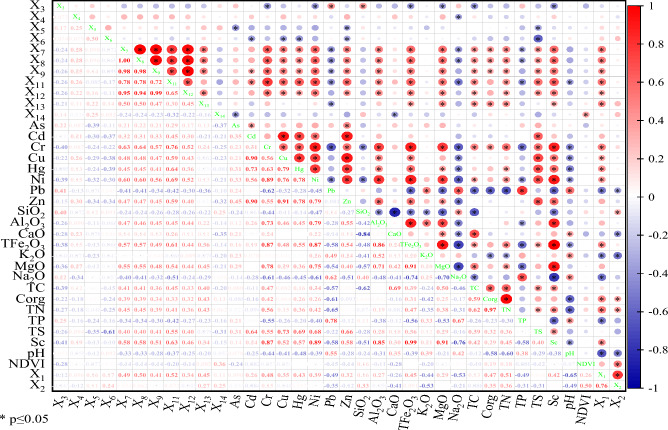
Pearson correlation coefficient of soil heavy metal elements and impact factors. Elevation (X_1_), slope (X_2_), aspect (X_3_), soil parent materials (X_4_), soil types (X_5_), soil erosion degree (X_6_), distance from railway (X_7_), distance from national highway G109 (X_8_), distance from county road (X_9_), distance from pastoral point (X_11_), distance from rural area (X_12_), distance from lake (X_13_), distance from river (X_14_).

The results of correlation analysis are shown in Fig. 4. The results showed that there was a significant correlation among most heavy metals, but interestingly, there was no significant correlation among As–Cu, As–Hg, As–Pb, Pb–Cr, Pb–Cu, Pb–Hg and Pb–Ni. Among the influencing factors of soil properties (SiO_2_, Al_2_O_3_, CaO, TFe_2_O_3_, K_2_O,MgO, Na_2_O, TC, C_org_, TN, TP, TS, Sc and pH), Cr, Cr, Ni and Zn were significantly correlated with 8–11 of them, and As and Hg were significantly correlated with 6 of them, among which As showed a weak correlation, ranging from − 0.26 to 0.27, Cd and Pb only had significant correlation with 4 and 3 of them, and except Cd–TS, the other correlations were weak, especially Pb had weak correlation with C_org_, TN and TP, which were easy to transfer and transform in soil. There was no significant correlation between NDVI and 8 heavy metals. Among the topographic factors (X_1_, X_2_ and X_3_), only Cr, Cu, Hg, Ni and Zn showed significant positive correlation with X_1_ and Ni-X_2_. In soil parent material (X_4_), soil type (X_5_) and soil erosion (X_6_), only Ni and Zn showed weak correlation with X_4_, Cd-X_5_ and Cd-X_6_. Among the distance factors (X_7_, X_8_, X_9_, X_11_, X_12_, X_13_ and X_14_), X_7_, X_8_ and X_9_ showed significant positive correlation with Cr, Cu and Hg, but significantly negative correlation with Pb. X_14_ showed no significant correlation with heavy metals. X_11_ showed significant positive correlation with Cd, Cr, Cu, Hg and Ni, but significant negative correlation with Pb. X_12_ showed a significant negative correlation with Pb and Zn, but a significant positive correlation with Cr, while X_13_ only had a significant positive correlation with Cr and Hg. Generally, As, Cd, Cr, Cu, Hg, Ni and Zn in 8 kinds of heavy metals are greatly affected by natural factors, but they are affected by some external sources, which is consistent with the previous research results^30,44^, but the heavy metal Pb is quite different, and its spatial variation may be caused by external sources.

### GeoDetector analysis of the factors influencing the spatial heterogeneity of heavy metals

#### Factor detection

**Figure 5:**
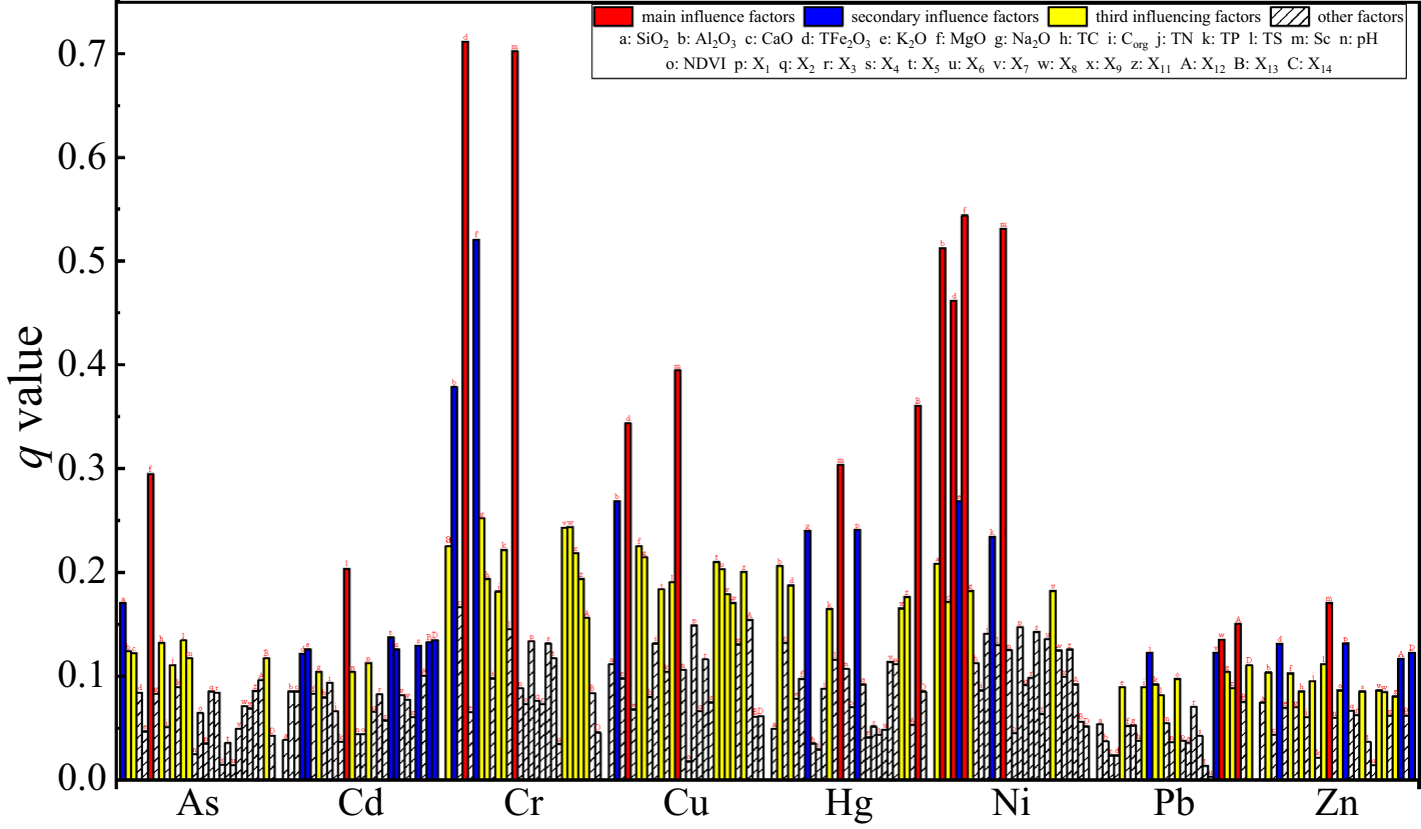
Effects of different factors on the explanatory power of eight heavy metals in soils with *q* value.

The explanatory power q value of 28 factors to 8 heavy metals detected by factor detector is shown in Fig. 5. There were significant differences in the explanatory power of different factors to 8 kinds of heavy metals. The main influencing factor of As is MgO, the q value is 0.295, the secondary influencing factor is SiO_2_ (0.170) and the third influencing factors are TS, TC, Al_2_O_3_, CaO, Sc, X_13_, and TN, with q values ranging from 0.134 to 0.110. The primary influencing factor of Cd is TS (0.204), followed by X_5_, X_14_, X_13_, X_11_, K_2_O, X_6_, and TFe_2_O_3_, with q values ranging from 0.137 to 0.122, and the third influencing factors are X_1_ (0.113), Na_2_O (0.104), and Sc (0.104). The first influencing factors of Cr are TFe_2_O_3_ (0.712) and Sc (0.703), the second influencing factors are MgO (0.521) and Al_2_O_3_ (0.379), and the third influencing factors are Na_2_O, X_8_, X_7_, SiO_2_, TP, X_9_, X_11_, TC, TN, and CaO, with q values ranging from 0.252 to 0.167. The primary influencing factor of Cu is similar to Cr, which are also TFe_2_O_3_ (0.344) and Sc (0.395), the secondary influencing factor is Al_2_O_3_ (0.269), and the third influencing factors are MgO, Na_2_O, X_5_, X_6_, X_11_, TS, TN, X_7_, and X_8_, with q values ranging from 0.225 to 0.171. The primary influencing factors of Hg are X_13_ (0.361) and Sc (0.304), followed by X_1_ (0.241) and Na_2_O (0.241), the third influencing factors are Al_2_O_3_ (0.206), TFe_2_O_3_ (0.188), X_11_ (0.176), X_9_ (0.165), and TP (0.165). The main influencing factors of Ni are MgO (0.544), Sc (0.532), Al_2_O_3_ (0.513), and TFe_2_O_3_ (0.462), the secondary influencing factors are K_2_O (0.269) and TP (0.234), the third influencing factor is SiO_2_ (0.208), X_7_ (0.182), Na_2_O (0.182), and CaO (0.172). The first influencing factors of Pb are X_12_ (0.150) and X_8_ (0.135), the second influencing factors are X_7_ and TN, with q values of 0.123, and the third influencing factors are X_14_, X_9_, NDVI, TP, C_org_, K_2_O, X_11_, and TS, with q values ranging from 0.110 to 0.082. The primary influencing factor of Zn is Sc (0.171), followed by X_1_, TFe_2_O_3_, X_14_, and X_12_, with q values ranging from 0.132 to 0.117, the third influencing factors are TS, Al_2_O_3_, MgO, TN, NDVI, X_7_, TC, X_4_, X_8_, and X_11_, with q values ranging from 0.112 to 0.081.

The order of influence degree of different influence factors on different heavy metals is different, which reveals the heterogeneity of different heavy metal change mechanisms. From the main influencing factors of heavy metals, except Pb, Zn and Hg, the other five heavy metals were mainly affected by soil properties, indicating that the spatial distribution characteristics of soil As, Cd, Cr, Cu, Ni and Zn in the study area were mainly affected by natural factors. It is interesting that the spatial distribution characteristics of Hg are most closely related to the distance from the lake (X_13_) and soil Sc content, as well as to altitude (X_1_). Correlation analysis shows a highly significant positive correlation (p < 0.01) between Hg-X_13_ and Hg-X_1_, indicating that soil Hg in the study area may be closely related to the long-distance transportation and sedimentation of Hg in the atmospheric circulation while being affected by the soil parent material. This is similar to many previous research conclusions^44–47^. The main factors affecting the spatial heterogeneity of soil Pb in the study area are the distance from the countryside (X_12_) and the distance from G109 (X_8_). The distance from the railway (X_7_) and the county road (X_9_) are also important factors affecting the spatial heterogeneity of soil Pb, which further shows that the spatial distribution of soil Pb in this area is mainly affected by human factors. Liu^44^ have also studied the content of heavy metals in typical grassland soils in Tibet and believe that Pb in topsoil may come from atmospheric deposition caused by traffic emissions and industrial point sources. Zhang^40^ pointed out that the concentration of heavy metal Pb in Tibetan soil decreased with the increase of distance from the road. Although the primary influencing factor of Zn is Sc, its q value of 0.171 is only 1.46 times of the q values of X_12_ (0.117), and only 1.99–2.11 times of the human factors X_7_ (0.086), X_8_ (0.085) and X_11_ (0.081).In addition, the correlation coefficient of Pb–Zn is as high as 0.832 (p < 0.01), so it can be inferred that Zn in the study area is affected by natural factors as well as human factors to a large extent. From the point of view of the primary, secondary and third influencing factors with the greatest explanatory power, the spatial differentiation of As, Cd, Cr, Cu, Ni and Zn in soil heavy metals in the study area is mainly caused by natural factors, but also affected by certain anthropogenic factors, which is basically consistent with the results of Pearson correlation analysis (Fig. 4). The spatial differentiation of Hg is affected by both natural factors and atmospheric deposition. The spatial distribution of Pb is mainly affected by anthropogenic factors.

#### Interaction detection

The composition and structure of soil are complex, and the spatial distribution and pollution of heavy metals are usually formed by many factors, so it is impossible for a single factor to affect the distribution and change of heavy metals^4^. Therefore, using the interaction detector to analyze the interaction degree of various factors on the spatial distribution of heavy metals is helpful to accurately judge the deep driving mechanism that affects the spatial distribution of heavy metals^48^.

**Figure 6:**
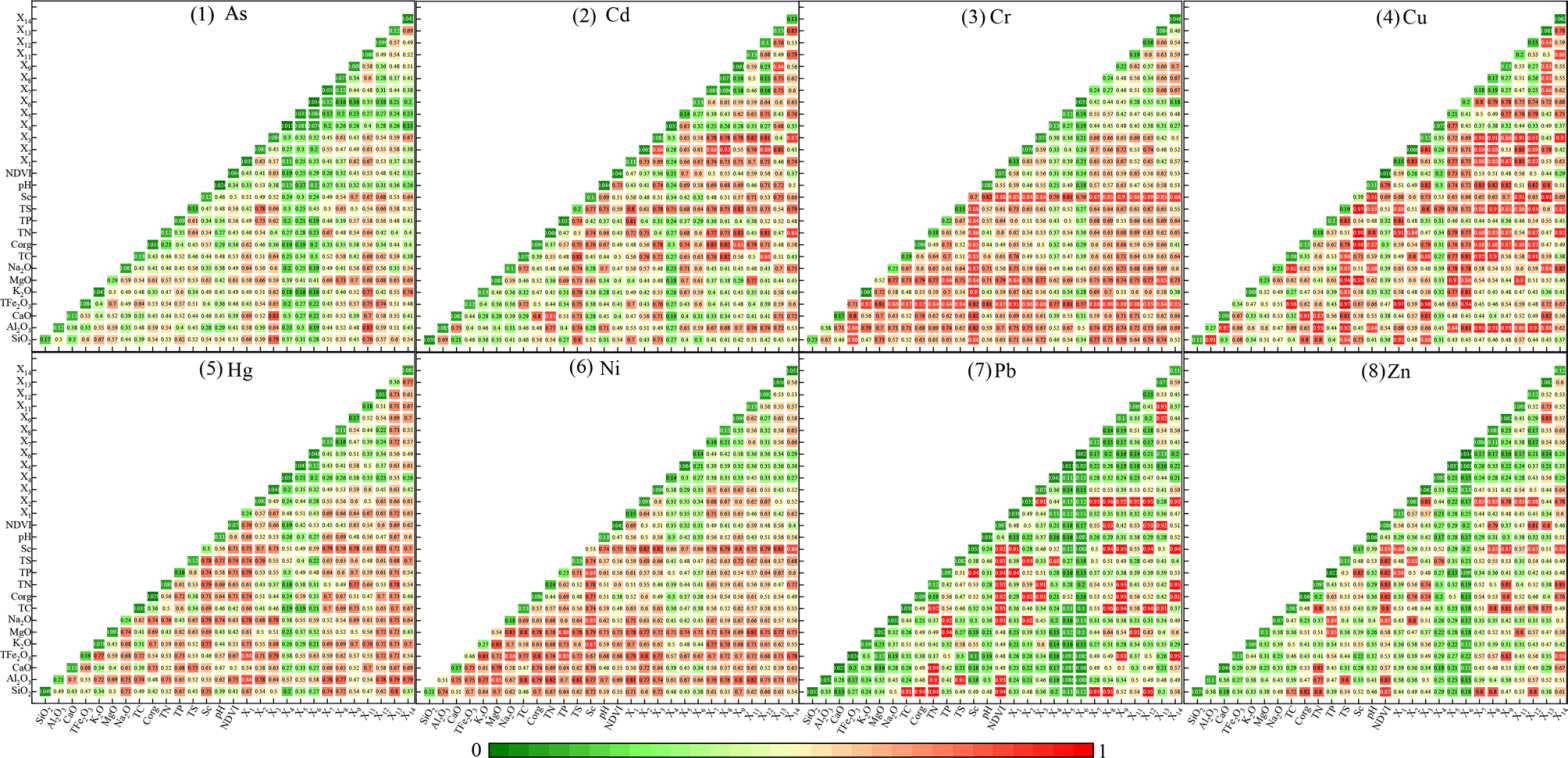
Interaction of different influence factors on soil heavy metals. Elevation (X_1_), slope (X_2_), aspect (X_3_), soil parent materials (X_4_), soil types (X_5_), soil erosion degree (X_6_), distance from railway (X_7_), distance from national highway G109 (X_8_), distance from county road (X_9_), distance from pastoral point (X_11_), distance from rural area (X_12_), distance from lake (X_13_), distance from river (X_14_).

The factor detection results show that the degree of explanation of the interaction of any two factors on the spatial differentiation of eight heavy metals is greater than that of a single factor, and most of them are nonlinear enhancement and a few are double factor enhancement, there is no weakening or independent type of action. For As is concerned (Fig. 6(1)), the strongest interactions are CaO ∩ X_3_, Al_2_O_3_ ∩ X_11_ and SiO_2_ ∩ X_3_, with q values of 0.830, 0.827 and 0.792, respectively, which are 2.68–2.81 times of the maximum factor detection q value of As (0.295). In addition, it can be seen that the distance from the herdsmen point (X_11)_ as a human factor also affects the distribution of soil As in this area. It may be caused by the long-term burning of yak manure and garbage incineration by local herdsmen^46,49^. For Cd (Fig. 6(2)), the interaction between TN ∩ X_14_ (0.881) and X_3_ ∩ X_14_ (0.875) is the strongest, approximately 4.3 times its maximum factor detection q value (0.204). In addition, CaO ∩ TN, TC ∩ TS, TC ∩ X_8_, TC ∩ X_12_, C_org_ ∩ X_7_, C_org_ ∩ X_8_, C_org_ ∩ X_9_, TN ∩ X_9_, TN ∩ X_12_, TP ∩ X_1_, TS ∩ X_1_, TS ∩ X_9_, X_3_ ∩ X_11_, X_3_ ∩ X_12_, X_9_ ∩ X_13_ and X_13_ ∩ X_14_ are all above 0.8, further indicates that although factors such as X_7_, X_8_ and X_9_ are not the main factors affecting the distribution of Cd in local soil, there is also a certain degree of influence. In addition, the strong migration, transformation and mobility of C, N, P, S with other influencing factors have a strong interaction on the Cd of the study area, which makes the migration mobility of Cd in this area is greater with the wetting of rain water, which is one of the possible reasons for the high Cd content of the Tuotuo river in the lower reaches of the region^39^. For Cr (Fig. 6(3)), the strongest interactions are TFe_2_O_3_ ∩ X_1_ (0.906), K_2_O ∩ Sc (0.898) and Sc ∩ X_9_ (0.897), compared with their maximum factor detection q value (0.712), the explanatory power q value is increased by about 126%. What is interesting is that most of the interactions between Sc and TFe_2_O_3_ and other influencing factors are above 0.8. Except for Sc and TFe_2_O_3_, the interaction between other factors on Cd was less than 0.8. For Cu (Fig. 6(4)), the largest q values of interaction are C_org_ ∩ Sc (0.978), TN ∩ Sc (0.976) and TFe_2_O_3_ ∩ X_3_ (0.976). Compared with their maximum factor detection q value (0.395), the explanatory power q value is increased by about 247%. In addition, the q value of X_7_, X_8_ and X_9_ interaction with other influencing factors is also more than 0.976. It can be seen that anthropogenic factors have a certain influence on the spatial differentiation of soil Cd in the study area. For Hg (Fig. 6(5)), the interaction of TFe_2_O_3_ ∩ X_1_, Al_2_O_3_ ∩ X_1_ and SiO_2_ ∩ X_13_ are the strongest, with q values of 0.840, 0.836 and 0.795, respectively, which are 2.20–2.33 times of their maximum factor detection q values (0.361). For Ni (Fig. 6(6)), the interaction between MgO ∩ TP (0.877), K_2_O ∩ Sc (0.868) and TFe_2_O_3_ ∩ Na_2_O (0.863) are the strongest, which is about 159% higher than its maximum factor detection q value (0.544). For Pb (Fig. 6(7)), the interaction between TC ∩ X_8_ (0.962), TC ∩ X_12_ (0.956) and X_2_ ∩ X_8_ (0.956) are the strongest, which is about 637% higher than its maximum factor detection q value (0.150). For Zn (Fig. 6(8)), the interaction between X_2_ ∩ X_12_ (0.875), SiO2 ∩ NDVI (0.868) and Sc ∩ X_9_ (0.867) are the strongest, which is about 508% higher than its maximum factor detection q value (0.171).

After careful observation, it was found that any two of the 28 influencing factors showed similar changes in the interaction between Pb and Zn, and the high or low values of q detected by the two heavy metals appeared in the interaction of the same factor pairs, which further confirmed the conclusion of factor detection that Zn was affected by natural factors as well as anthropogenic factors to a great extent. Generally, although the explanatory power q values of human factors in factor detection are relatively small, through the interactive detection results, it can be found that the interaction of these anthropogenic factors and other factors has an important impact on the spatial differentiation of heavy metals in this region. The interaction of various factors can better explain the spatial heterogeneity of heavy metals and provide interesting information.

#### Risk detection

**Figure 7:**
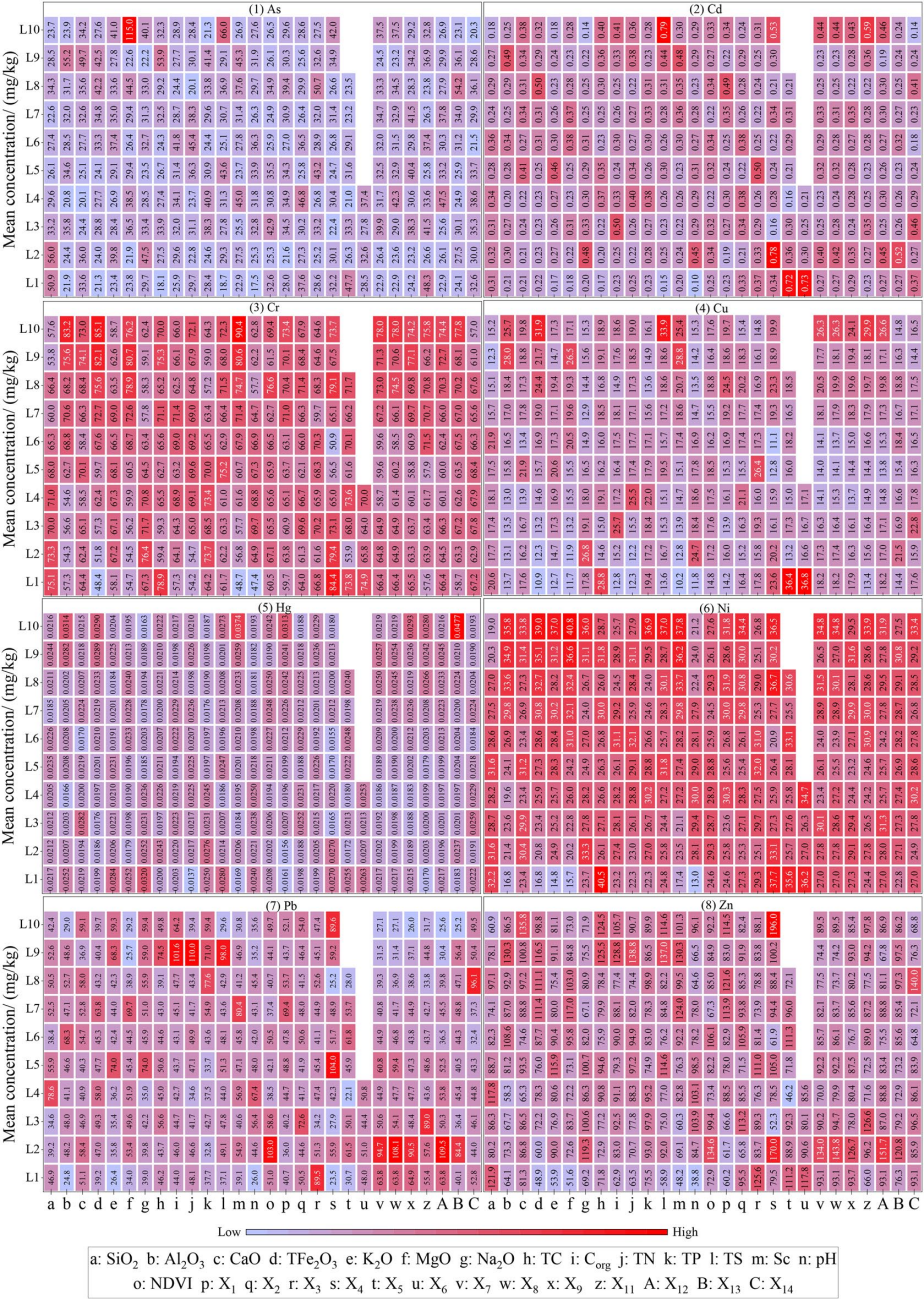
Risk detection of heavy metals content.

The risk detector was used to detect the significant difference of heavy metals between the two sub-regions of 28 factors and the high value area of heavy metals in each factor sub-region (Fig. 7). The significant differences of different heavy metals in different influencing factors are different, taking the influencing factor SiO_2_ as an example. For As (Fig. 6(1)), its average content 56.0 mg/kg in the SiO_2_ sub region L2 (49.14–53.8%) is the highest, and there are significant differences between L2 with L4, L5, L6, L7 and L10, as well as L3 with L7. For Cd (Fig. 6(2)), its average content 0.36 mg/kg in the SiO_2_ sub region L6 (60.38–61.95%) is the highest, with only significant differences between L10 with L3 and L4; For Cr (Fig. 6(3)), its average content 75.1 mg/kg in the SiO_2_ sub region L1 (< 49.14%) is the highest, and there are significant differences between L1 with L4, L5, L6, L7, L8, L9 and L10, L7 with L2, L3, L4 and L5, L9 with L2, L3, L4, L5, L6, L7 and L8, as well as L10 with L2, L3, L4 and L5; For Cr (Fig. 6(4)), its average content 21.9 mg/kg in the SiO_2_ sub region L6 (60.38–61.95%) is the highest, and there are significant differences between L1 with L3, L4, L5, L7, L8, L9 and L10, L9 with L2, L3, L4, L5, L6, L7 and L8, as well as L4 with L8; For Hg (Fig. 6(5)), its average content 0.0244 mg/kg in the SiO_2_ sub region L9 (66.95–70.88%) is the highest, with only significant differences between L9 with L7; For Ni (Fig. 6(6)), its average content 32.2 mg/kg in the SiO_2_ sub region L1 (< 49.14%) is the highest, and there are significant differences between L1 with L2, L3, L4, L5, L6, L7, L7, L8, L9 and L10, L10 with L2, L3, L4, L5, L6, L7, L8 and L9, as well as L9 with L3 and L4; For Pb (Fig. 6(7)), its average content 78.6 mg/kg in the SiO_2_ sub region L4 (56.24–58.29%) is the highest, with only significant differences between L3 with L5, L7 and L8, and L6 with L7; For Zn (Fig. 6(8)), its average content 121.9 mg/kg in the SiO_2_ sub region L1 (< 49.14%) is the highest, with only significant differences between L10 with L1, L3, L5, and L8.

According to the 28 factors, the average content of As was the highest in the sub-region where the MgO < 2.46% (L10), the average content of 115 mg/kg; Cd was the highest in the L10 sub-region of TS (> 504 mg/kg), at 0.79 mg/kg; Cr was the highest in the L10 sub-region (> 12.6 mg/kg) of Sc, which was 90.4 mg/kg. The average content of Cu was the highest in the L1 sub-region (no erosion) of the influence factor X_6_, which was 36.8 mg/kg. The average content of Hg in the L10 sub-region of influence factor X_13_ (> 7103 m) is the highest, which is 0.0477 mg/kg. The average content 40.8 mg/kg of Ni is the highest in the sub-region where the influence factor MgO content is more than 2.46% (L10). The average content 110.0 mg/kg of Pb is the highest in the L9 sub-region (1394–1739 mg/kg) of the influencing factor TN. The average content 196.0 mg/kg of Zn in the L10 sub-region (slope and alluvial parent materia) of influence factor X_4_ was the highest. In addition, the analysis shows that the results of risk detection are consistent with the results of factor detection, that is, factor detection has great explanatory power on heavy metals, and there are significant differences in the content of heavy metals among their subregions.

#### Ecological detection

Ecological detection focuses on comparing whether there is a significant difference between one influence factor and another influence factor on the spatial distribution of soil heavy metals^4^, if significant, it will be recorded as Y, otherwise it will be recorded as N.

**Figure 8:**
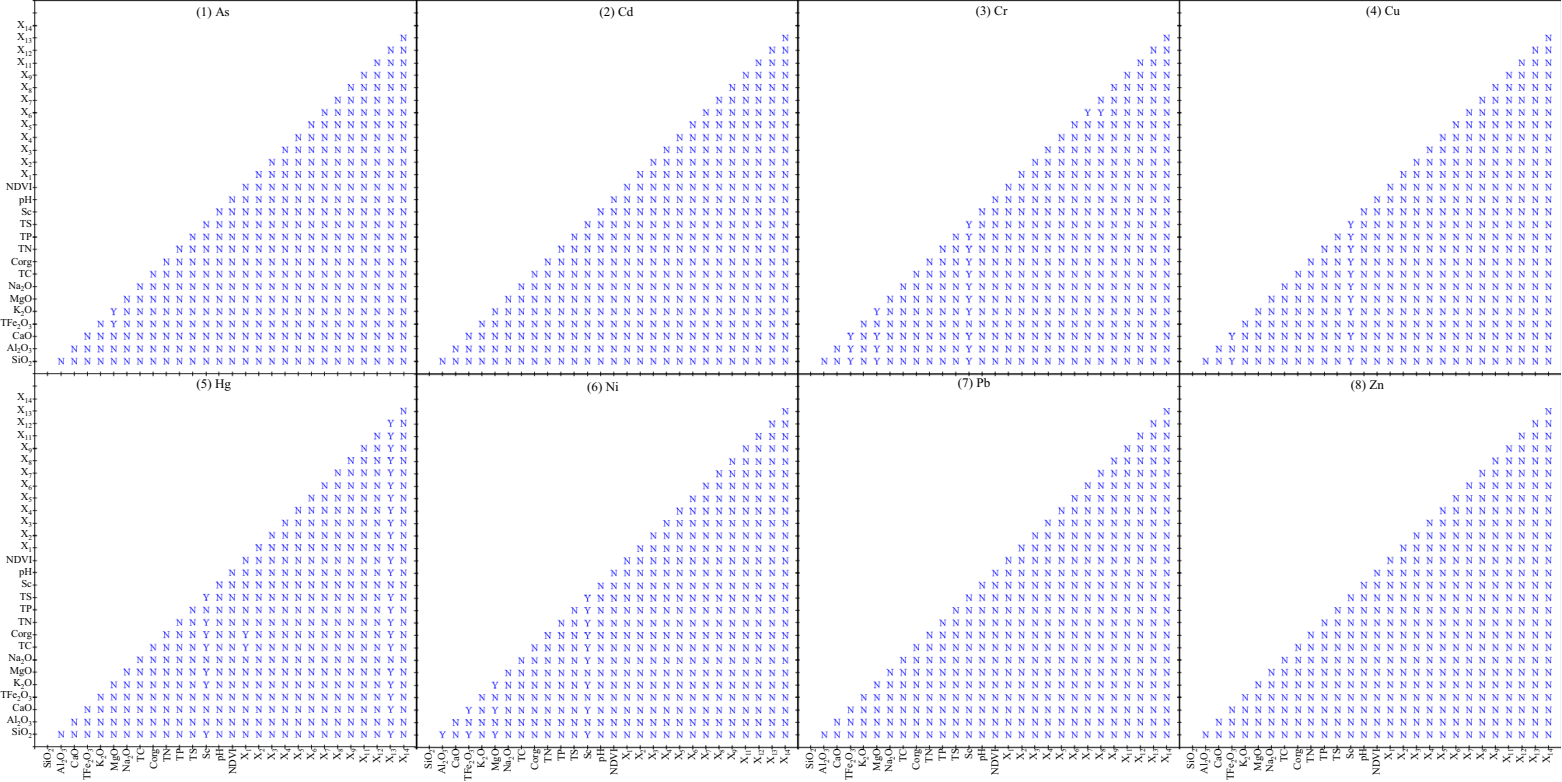
Ecological detection results of soil heavy metals in study area.

The ecological detection results of soil heavy metals in the study area showed that there were significant differences in the effects of MgO with TFe_2_O_3_ and K_2_O on As (Fig. 8(1)), but there were no significant differences among other factors. There are significant differences in the effects of TFe_2_O_3_ with SiO_2_, Al_2_O_3_ and CaO, MgO with SiO_2_, Al_2_O_3_, CaO and K_2_O, Sc with SiO_2_, Al_2_O_3_, CaO, TFe_2_O_3_, K_2_O, MgO, Na_2_O, TC, C_org_, TN, TP and TS, and X_6_ with X_7_ and X_8_ on Cr (Fig. 8(3)), but there are no significant differences among other factors. There were significant differences in the effects of TFe_2_O_3_ with SiO2 and CaO and Sc with SiO_2_, CaO, K_2_O, MgO, Na_2_O, TC, C_org_, TN, TP and TS on Cu (Fig. 8(4)), but there were no significant differences among other factors. The effects of Sc with SiO_2_, K_2_O, MgO, Na_2_O, TC, C_org_, TN, TP and TS, X_1_ with TC and C_org_, and X_13_ with SiO_2_, CaO, TFe_2_O_3_, K_2_O, MgO, TC, C_org_, TN, TP, TS, Sc, pH, NDVI, X_2_, X_3_, X_4_, X_5_, X_6_, X_7_, X_8_, X_9_, X_11_ and X_12_ on Hg were significantly different (Fig. 8(5)), but there were no significant differences among other factors. There were significant differences in the effects of Al_2_O_3_ with SiO_2_, TFe_2_O_3_ with SiO_2_ and CaO, MgO with SiO_2_, CaO and K_2_O, and Sc with SiO_2_, CaO, K_2_O, Na_2_O, TC, C_org_, TN, TP and TS on Ni (Fig. 8(6)), but there were no significant differences among other factors, and there were no significant differences among the 28 influencing factors of Cd (Fig. 8(2)), Pb (Fig. 8(7)) and Zn (Fig. 8(8)).

## Conclusions

1. The average contents of Hg, Cr, Cu and Ni in the study area are lower than the background values of Tibetan soil, but the average contents of As, Cd, Pb and Zn are 1.62, 3.64, 1.69 and 1.20 times of the background values, respectively. The coefficients of variation of As, Cd, Pb and Zn were all more than 65%, and the average content of As was higher than the soil pollution risk screening value (GB15618-2018), while the average values of the other seven heavy metals were lower than the soil pollution risk screening value. The geoaccumulation index showed that AS, Cr, Cu, Ni, Hg and Zn were in clean state, while Cd and Pb were slightly polluted; enrichment factors showed that the total amount of Cr, Cu, Hg, Ni and Zn was slightly enrichment, while As, Cd and Pb were moderately enrichment; pollution index showed that As was in low pollution state, and the other seven heavy metals were safe.
2. There are significant differences in the spatial distribution of soil heavy metals in the study area. The high value areas of As are mainly concentrated in the central and southern regions of the study area, the high value areas of Cd are concentrated in the western and southern regions, the high value areas of Cr are concentrated in the northwest and southwest regions, the high value areas of Cu are mainly distributed in the western region, the high value areas of Hg are mainly distributed in the southwest region, and the high value areas of Ni are concentrated in the northwest, central and southern regions. The high value areas of Pb and Zn are concentrated in the southern region.
3. Correlation analysis showed that most of the eight heavy metals had significant correlations. Soil heavy metals had the strongest correlation with soil properties, followed by distance factors, and relatively weak correlations with topographic factors, soil parent materials, soil types and soil erosion, but no correlation with NDVI.
4. The detection results of 8 heavy metals and 28 influencing factors show that the spatial distribution characteristics of As, Cd, Cr, Cu and Ni are mainly affected by natural factors. The spatial distribution characteristics of Hg are most closely related to the distance from the lake (X_13_) and the content of soil Sc. The spatial heterogeneity of Pb is mainly affected by the distance from the countryside (X_12_) and the distance from G109 (X_8_). The spatial distribution characteristics of Zn are not only affected by natural factors, but also by human factors. Interactive detection found that the interaction explanatory power all showed enhanced effect, and the strongest interaction explanatory power q value of 8 heavy metals increased by 126%, 637% compared with their respective strongest factor detection explanatory power q value. The interaction of human factors and other factors has an important impact on the spatial differentiation of heavy metals in the study area. Risk detection showed that there were significant differences among different influencing factors. As, Cd, Cr, Cu, Hg, Ni, Pb and Zn had the highest average content of heavy metals in MgO (L10), TS (L10), Sc (L10), X_6_ (L1), X_13_ (L10), MgO (L10), TN (L9) and X_4_ (L10), respectively. The difference of heavy metals content among its sub-regions is particularly significant. Generally, the spatial distribution of soil heavy metals in the study area is the result of many factors, and the effects of different factors on different heavy metal elements are different. The spatial differentiation of As, Cd, Cr, Cu, Ni and Zn is mainly caused by natural factors, but there are also some anthropogenic factors. The spatial differentiation of Hg is affected by both natural factors and atmospheric deposition. The spatial distribution of Pb is mainly affected by anthropogenic factors.

In general, the spatial distribution of soil heavy metals in the study area was mainly affected by natural factors, but some heavy metals (such as Hg, Pb and Zn) were also affected by human factors. Further studies are needed to provide a basis for strengthening the “source control” of heavy metal pollution.

## Data Availability

The authors declare that all data supporting the findings of this study are available within the article.
